# Supplementary material for: On the physical origin of linguistic laws and lognormality in speech
Source: R Soc Open Sci. 2019 Aug 21;6(8):191023. doi: 10.1098/rsos.191023 (PMC6731709; doi:10.1098/rsos.191023)
Supplement: Additional Results and details of "On the physical origin of linguistic laws and lognormality in speech" [file rsos191023supp1.pdf]

Supplementary Information for "On the physical origin of linguistic laws and lognormality in speech"

Iván G. Torre[1,2,*], Bartolo Luque[1], Lucas Lacasa[3], Christopher T. Kello[2] and Antoni Hernández-Fernández[4,*]
[1] *Departamento de Matemática Aplicada, ETSIAE, Universidad Politécnica de Madrid, Plaza Cardenal Cisneros 28040 Madrid (Spain)*
[2] *Cognitive and Information Sciences, University of California, Merced, 5200 North Lake Rd. Merced, CA 95343 (United States)*
[3] *School of Mathematical Sciences, Queen Mary University of London, Mile End Road E14NS London (UK)*
[4] *Complexity and Quantitative Linguistics Lab, Laboratory for Relational Algorithmics, Complexity and Learning (LARCA); Institut de Ciències de l'Educació; Universitat Politècnica de Catalunya, Barcelona (Spain)[*]*

Contents

I. Additional details on the Buckeye corpus 2

II. Additional results on the stochastic model of time duration 2
A. Lognormality law for individual speakers 2
B. Additional representations on the duration distribution of linguistic units 4
C. Limit distributions of sums of independent Lognormals 4
D. The error terms at word and BG levels 6

III. Zipf's law: additional details and results 6
A. Model selection scheme for Zipf's law: BIC 6
B. A null model for Zipf's law 6
C. Zipf's law for individual informants 7

IV. Herdan's law: additional evidence based on speech rate 8

V. Binning: additional details 8
A. Binning in scatter plots with high noise in one of the variables 8
B. Binning in frequencies 9

VI. Menzerath-Altmann law: additional results 9
A. Additional fits of MAL 9
B. Predicting BG time duration distribution using the MAL model 10

References 11

[*] Electronic address: ivan.gonzalez.torre@upm.es, antonio.hernandez@upc.edu

I. ADDITIONAL DETAILS ON THE BUCKEYE CORPUS

While exploration of linguistic laws was typically carried out in small and fragmented corpora, in recent years an important transition is taking place in the core of linguistics, where more scholars are adopting tools and methodologies coming from natural sciences [1, 2]. In this sense, corpus linguistics has become a key area which provides extensive empirical data that enables analysis beyond the study of isolated phrases and small datasets with few informants as well as the development of theoretical models and more formal approaches to the study of language [3].

For the phonetic labeling, 64 symbols were used in line with the traditional phonetic models of American English [4]: 41 phonemes plus 23 phonetic variations including flaps, stops, and nasals. The phonetic segmentation and labeling of the corpus was carried in two phases: first it was automatically labeled an aligned and then it was corrected by trained transcribers with the help of the audio signal, speech wave and spectrograms. While numeric resolution of phonetic alignment is below 1 ms, the consistency in labeling and segmentation of words and phonemes was analyzed by comparing the same sample transcription of six Buckeye transcribers. It was found that the mean deviation in boundary placement difference across all transcribers was 16 ms, having larger consistency for longer phonemes and words [5]. Although it is not possible to establish an error of precision due to the lack of a ground truth, we interpret the consistency analysis as a reference of the lowest reliable resolution, given that the total glottal pulse length is reported to be about 10 ms or slightly higher [4], the lower bound uncertainty is close to the physiological limit and wholly appropriate for this study. All those details are further explained in Buckeye Corpus Manual [6].

We have statistically characterized the durations of phonemes, words and BGs of an extensive corpus (Table I of the main manuscript). The values obtained are in the order of magnitude expected for the measures of central tendency although with some peculiarities, that should be associated to the fact of working with an oral corpus[7]. Thus, for example, if the classic work of Shannon [8] worked with an average word length in English of 4.5 letters, to determine an entropy per word of 2.62 bits, here we have determined a near mean (and median) of 4 ± 2 letters per word. It could also be said that the mean (and median) values obtained for phonemes (Table I in the main manuscript) are in the order of magnitude expected for good intelligibility and acoustic perception [9, 10], as it could not be otherwise, so that the communication is successful.
On the other hand, as a limitation, the extension of some outliers of the breath groups suggests that the speakers have caught air in the middle of their pronunciation, so physiologically they would not be strictly considered BG. This fact might have slightly influenced the BG statistics.

The average durations of BGs have been previously explored in American English in both spontaneous speech and in reading [11], with mean durations somewhat higher than those obtained here for the Buckeye Corpus (we found 1.4 ± 1.2 s versus 3.50 ± 0.62 s in passage reading and 4.35 ± 0.72 s in spontaneous speech in [11]), which may indicate that the speakers of Buckeye corpus speak relatively faster than in these previous studies or a significant difference in segmentation methods [11]. In addition, the relevance of stress accent in the duration of the syllables has also been focused in other studies [7]: the importance of the stress accent in the duration of the syllables has been demonstrated, as well as the number of syllables and their structure within each word, which finally influences word duration. However, the mean duration of the words in this previous study of American English coincides with the one found here (0.257 s in [7] and 0.24 ± 0.17 s here, with median 0.2). Finally, with regard to the mean duration found for phonemes we can say that it is a value in which multiple known factors collapse, as is the case of the relative frequency of each phoneme [12] or the influence of prosody and phonemic context on the duration of consonants [13] and vowels [14] (see SI for additional details and comments).

II. ADDITIONAL RESULTS ON THE STOCHASTIC MODEL OF TIME DURATION

A. Lognormality law for individual speakers

Here we show evidence that duration distribution of phonemes, words and BG are also lognormal distributed when studying individual speakers. For this purpose we have chosen the first 9 informants of Buckeye corpus and we report in Figure S1 time duration distribution of phonemes, words and BG. As it was shown for the entire database, duration distribution for all linguistic levels agree with LND.

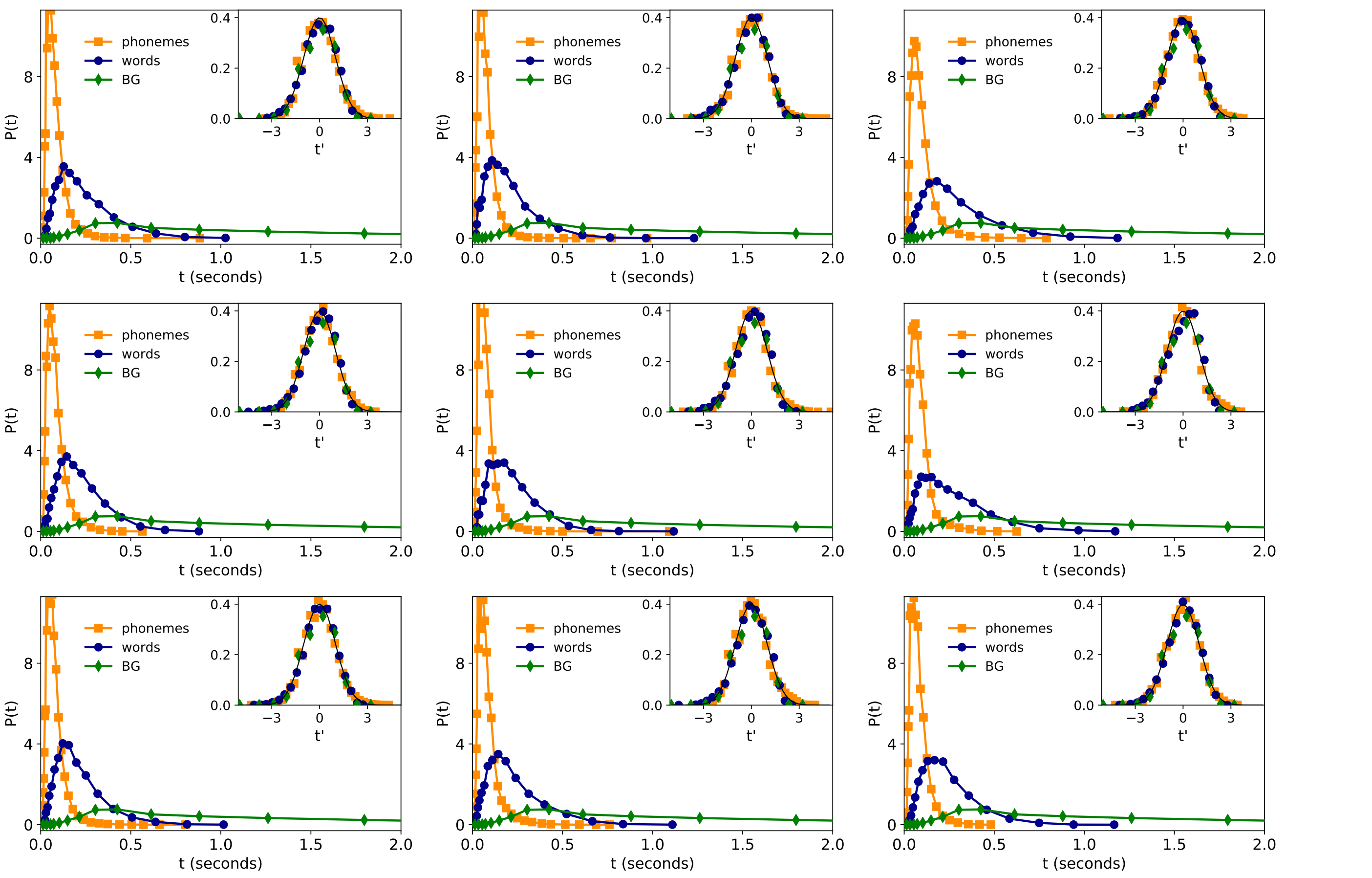

Figure S1: **Lognormality law for individual speakers** Time duration distribution for the first 9 informants of the Buckeye corpus. (Outer panels) Estimated time duration distribution of breath groups (green diamonds), words (blue circles) and phonemes (orange squares) for each informant (a logarithmic binning has been applied to the histograms, and solid lines are guides for the eye). (Inner panels) We check the validity of the lognormal hypothesis by observing that, when rescaling the values of each distribution $t' = \frac{\log(t) - \langle \log(t) \rangle}{\sigma(\log(t))}$, all data collapse into a universal standard Gaussian (solid black line is $\mathcal{N}(0,1)$).

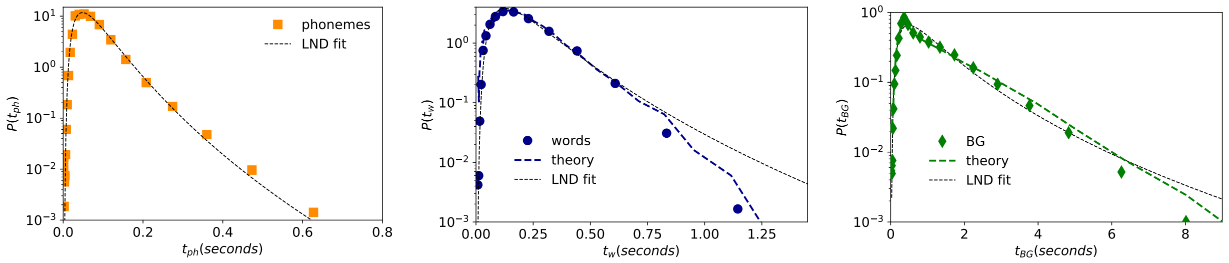

Figure S2: **Duration distribution of linguistic levels.** From left to right: linear-log representation of phonemes, words and BG time duration distribution. Orange squares, blue circles and green diamons corresponds to the empirical time duration distribution. Black dotted line is a maximum likelihood estimation (MLE) fit to a lognormal distribution. Coloured dashed lines are the theoretical prediction of the stochastic model (see main text for details). Note that the stochastic model visually agree better the tails of the distribution than the LND.

B. Additional representations on the duration distribution of linguistic units

In order to observe more closely the tails of the distribution, in Figure S2 we report time duration distribution for all phonemes, words and BG using log-linear axis. We find that while for the case of phonemes LND fits well all data, in the case of words and BG, the tail of the distribution is better explained using the stochastic model than the LND model.

C. Limit distributions of sums of independent Lognormals

The limit distribution of a sum of n independent Lognormals $Z = \sum_{i=1}^{n} Y_i$ (when n is small) is in general not known, and can vary with the parameters of the underlying Lognormal distributions and the underlying distribution of n (see panel (h)). However, in a large number of cases, such distribution is well approximated by a Lognormal distribution provided each Y_i follows a Lognormal distribution with reasonably similar parameters. A numerical confirmation is shown in panel (a) of Fig.S4. When n itself is a random variable the casuistic is in principle larger, but results seem to be similar provided that the variable $n = 1$ is underrepresented (panels (b) and (c) of the same figure). When these hypothesis are not met, the limit distribution tends to deviate from a Lognormal distribution (panels d-e). In the limit $n \to \infty$, the central limit theorem enforces a Gaussian as the limit distribution of the sum (panel f). Note that convergence towards CLT is not achieved when n is small (panel (g)).

When we combine a fixed number of phonemes n to construct a word following the model $Z = \sum_{i=1}^{n} Y_i$, the resulting time duration is Lognormal (see left panel of Fig. S3. However in order to be quantitatively accurate n cannot be fixed but needs to be a random variable which we sample from the actual $P(n)$. A similar phenomenon occurs at BG level (right panel of the same figure).

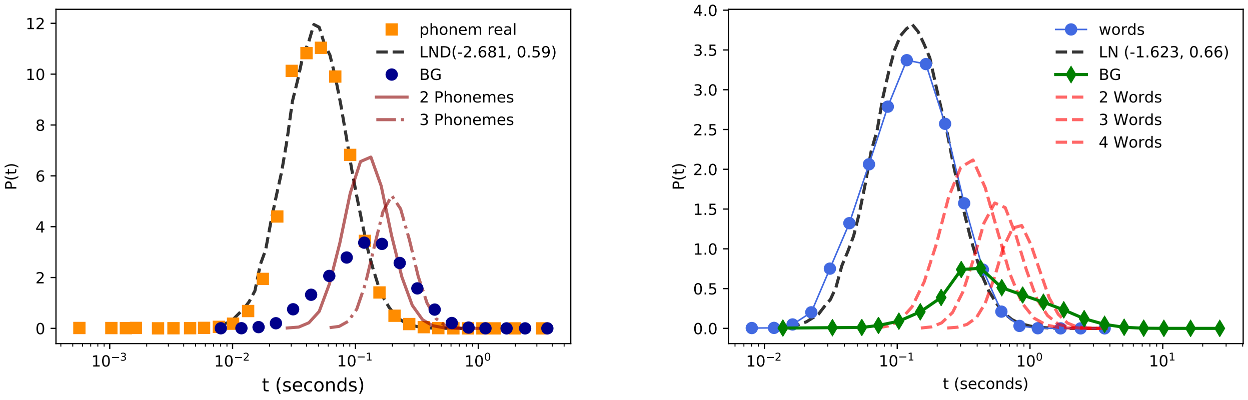

Figure S3: n **needs to be a random variable.** (Left panel) Linear-log plots of time distributions of phonemes and words. When we fix $n = 2$ or $n = 3$, the sum of n Lognormals (where each sample is extracted from the Lognormal fit to the phoneme distribution with parameters $\mu = -2.68$ and $\sigma = 0.59$ (gray dot line) is indeed Lognormal, but the resulting variable distribution (red dashed curves) is not in quantitative agreement with the actual word time distribution (blue curve). (Right panel) Similar phenomenon at the BG level. When we sample a fixed number of words n, the resulting time distribution of BG is Lognormal, but deviates from the actual one.

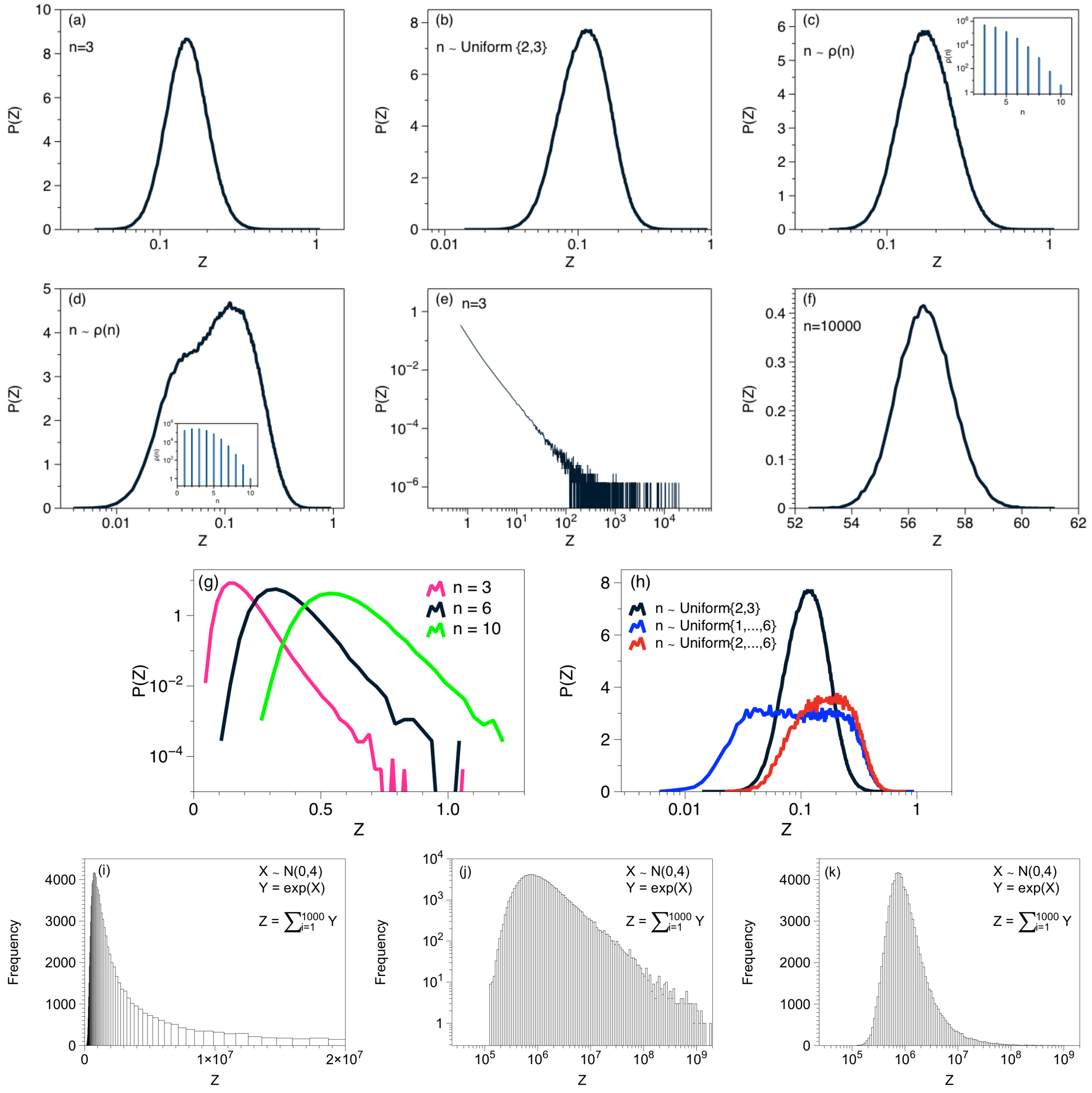

Figure S4: **Sum of lognormals: taxonomy.** Log-linear plot of the estimated time duration distribution $P(Z)$ of (a) the sum of $n = 3$ random variables extracted from similar Lognormal distributions, $Z = \sum_{i=1}^{3} Y$. Each random variable $Y = \exp(X)$ where $X \sim N(\mu, \sigma)$ (i.e. Y is Lognormal) such that $\mu = -3 + \xi_1, \sigma = 0.5 + \xi_2$, with $\xi_1, \xi_2 \sim N(0, 0.05)$; (b) Same as panel (a) but where n is now itself a random variable sampled from a Uniform distribution $U\{2, 3\}$; (c) Same as panel (b) but where n is sampled from a distribution $\rho(n)$ whose histogram is depicted in the inner panel. In all these cases the sum Z approaches a Lognormal distribution.
Panel (d) is same as panel (c) but where n is sampled from a distribution $\rho(n)$ whose histogram is depicted in the inner panel, where $n = 1$ is overrepresented. We observe a deviation from Lognormality. In Panel (e) we set $n = 3$, and each random variable Y_i is extracted from very different Lognormal distributions with very large variances: $Z = \sum_{i=1}^{3} Y, Y = \exp(X)$ where $X \sim N(\mu, \sigma)$ (i.e. Y is Lognormal) such that $\mu = -3 + \xi_1, \sigma = 0.5 + \xi_2$, with $\xi_1, \xi_2 \sim N(0, 1)$. The plot is log-log in this case, hence Z appears power-law distributed. Panel (f) is similar to panel (a), but with $n = 1000$ random variables, and the plot is in linear scales. Central limit theorem kicks in and the limit distribution of the sum is Gaussian. (g) Similar to panel (a) but the plot is log-linear (a normal distribution would look like an inverted parabola in this plot). For increasing n the lognormal character of its sum remains, i.e. convergence to CLT is slow. (h) Similar to panel (b), but for n being a random variable extracted from various unform distributions. The lognormal shape is easily broken down. (Panels (i-k)) Frequency histogram of the sum of 1000 i.i.d lognormal random variables Y (10^5 samples of Y where computed). For this case, CLT has not kicked in yet as $Z = \sum Y$ is clearly non-Gaussian (panel i). The sum actually seems to be still well approximated by a lognormal distribution, although panel (k) reveals that this approximation is not very good as this plot is skewed (a lognormal distribution on linear-log scale should appear Gaussian and thus symmetric).

D. The error terms at word and BG levels

It is sensible to assume that there exist small segmentation errors at any level. However, note that there is a fundamental difference between what happens at phoneme or word level and at BG level. In the former scenarios, the source of error is expected to be of zero mean, simply because if one element is segmented larger than it should be, this bias is then substracted from the adjacent element. On the other hand, this cancellation is not present in the case of BGs. At the beginning and end of a BG, any error in the segmentation is not counterbalanced because BGs are separated by silences. These silences act as safe gaps and allow the segmenter to conservatively locate the beginning and end of each BG (the heuristic being try to always include all BG signal, at the expense of sometimes including part of the adjacent silence [5, 6]).

The explanation above, also briefly discussed in the main manuscript, suggests that the experimental records of BG time durations are systematically polluted by a measurement error with (small but) positive mean, something we take into account in our model. We should also mention different sources that make the segmentation of speech signal vs silence –i.e. the location of the beginning and end of BGs– difficult. These include (i) the phenomenon of coarticulation, or the variation that a speech sound undergoes under the influence of neighbouring sounds that can occur in different temporal levels [15], and (ii) Voice Onset Time (VOT), defined as the difference between the time of the burst and the onset of voicing in the following vowel that usually happens in plosives and sometimes at the beginning of phonation [4].

The mean of the Gaussian error which is systematically added in our model to match the empirical BG time distribution is plausably consistent with a mixed effect coming from a VOT and a conservative segmentation.

III. ZIPF'S LAW: ADDITIONAL DETAILS AND RESULTS

A. Model selection scheme for Zipf's law: BIC

In order to distinguish whether a double power law is a better fit than a single power law in the case of Zipf's law, and besides arguing from a linguistic point of view that the former is more sensible than the latter in the corpus considered here, we have performed a statistical model selection, based on minimizing the Bayesian Information Criterion (BIC)

$$\mathrm{BIC} = \ln(N)k - 2\ln(\mathcal{L}),$$

where N is the number of samples, k is the number of parameters of the model, and $\mathcal{L}$ the likelihood function. Since statistical models with more parameters tend to reach higher likelihood (i.e. because of overfitting), BIC penalize the increase of complexity in the model. The likelihood function is defined as:

$$\mathcal{L}(\theta \,|\, x_1, \dots, x_n) = \prod_{i=1}^{n} f(x_i|\theta).,$$

so the log-likelihood is just $\ln \mathcal{L} = \sum_{i=1}^{n} \ln f(x_i|\theta)$, where x_i are the data points and θ the estimated parameters of the model. When we apply this method to the empirical frequency-rank plot of words, we find BIC values of $3.34 \cdot 10^6$ for the (single) power law fit and $3.27 \cdot 10^6$ for the double power law fit. The difference is substantial, concluding that the double power law fit is also preferred statistically.

B. A null model for Zipf's law

One of the classic criticisms of statistical models in language is about the fact that Zipf's law can be retrieved randomly in models of so-called *intermittent silence* or *monkey typing* [16, 17]. Although these models have been duly refuted [18], here we propose a null model that certifies that in the acoustic study of language –not only in texts– Zipf's law is still not recovered by chance.

As is traditional in corpus linguistics [19], let us define linguistic elements as *type* and let us define each particular instance of a given type as a *token*. Let us create n bins between the minimum and maximum duration of words. Accordingly, each 'word' here is defined to be the same type if its duration holds within the limits of the same bin. This would be equivalent to reorder by frequency the lognormal distribution of Figure 2 in the main manuscript,

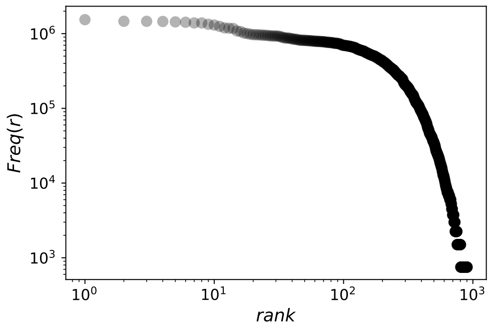

Figure S5: The null model does not follow Zipf's law, certifying that such statistical law does not occur 'by chance'.

substituting the values of x-axis by its rank. The result of doing this does not follow Zipf's law and it is shown in Figure S5. This is a new evidence against explanations of Zipf's law based on random typing [16, 17, 20] previously already contrasted [18].

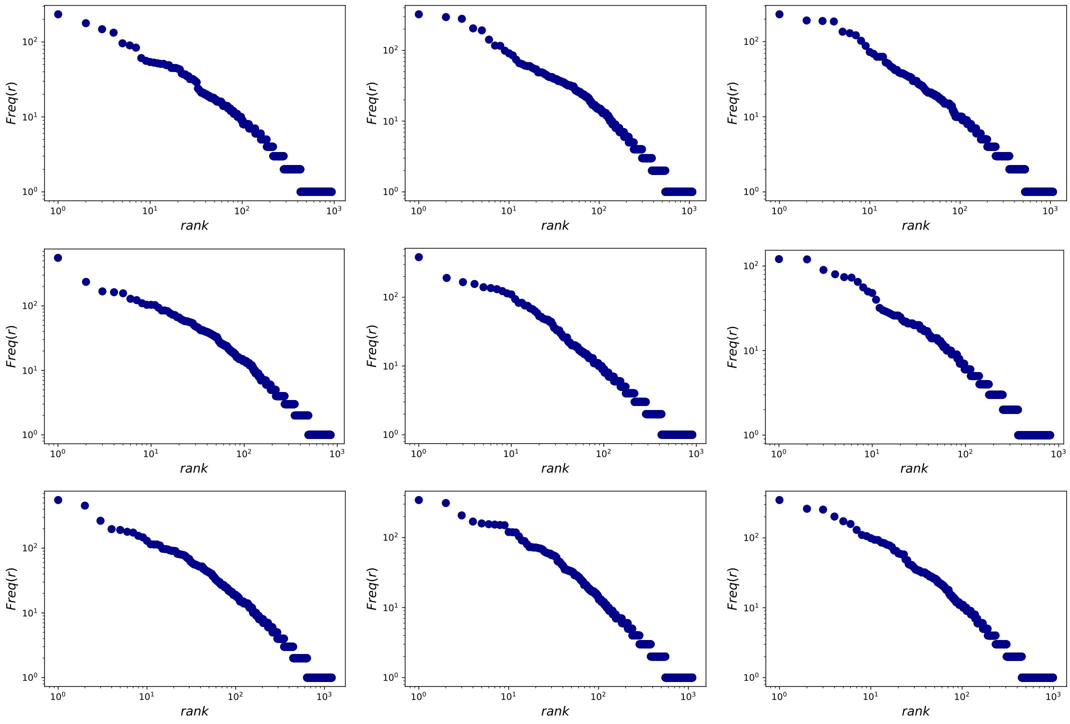

Figure S6: Zipf's law for 9 individual informants of Buckeye corpus. The plots suggest that each Zipf's law for each speaker could be fitted by a simple power law with an x_{min}.

C. Zipf's law for individual informants

In order to clarify the origin of the power law with two regimes found when studying the Buckeye Corpus, we also analyze Zipf's law for each individual informant and represent 9 of them in Figure S6. At the naked eye, the plots suggest that the rank-frequency law for each individual informant might be explained with a simple power law with an x_{min} (obtaining different x_{min} for each speaker), and this would agree with [21] in which the emergence of double power law scaling is due to mixing effects of multi-author corpus. This possibility is not however totally clear, and in order to conclude whether a pure power law model with cut-off or a double power law model is preferred for individual speakers, a model selection analysis should be done for each informant. This analysis goes beyond the

scope of this paper and we thereby remain agnostic, while acknowledging that multi-speaker mixing might be causing the emergence of double power law scaling.

IV. HERDAN'S LAW: ADDITIONAL EVIDENCE BASED ON SPEECH RATE

In results section C of the main manuscript, we mathematically justify that Herdan's law holds in physical and symbolic units with the same exponent since the number of words elapsed L results to be proportional to the average time T of the conversation elapsed after L words, i.e. $T \propto L$. Here we empirically validate this finding. In Figure S7 we plot the speech rate L/T (number of words per minute) as a function of L, and find that such quantity after a transient converges to a constant value. This evidence supports the analysis shown in the main text where the slope of the stable regime of Herdan's law has to be the same for oral and written magnitudes.

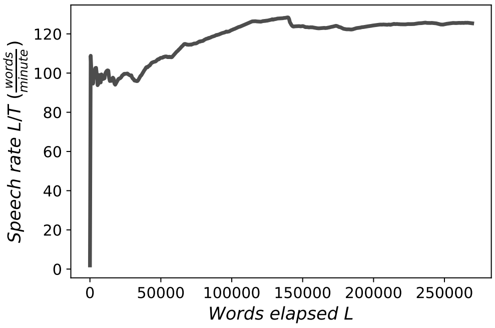

Figure S7: Plot of speech rate L/T (in number of words per minute) as a function of the number of words elapsed. After a transient, the speech rate reaches a value of about 125 words/minute, and maintains constant thereafter along the rest of the corpus, hence finding that $L \propto T$, in good empirical agreement with the mathematical derivation proposed in results section C of the main text.

V. BINNING: ADDITIONAL DETAILS

A. Binning in scatter plots with high noise in one of the variables

Data binning is a useful statistical technique which helps to reduce noise and finite size effects in a two-dimensional plot (x, y). Binning is performed by grouping the original data into bins. For instance, if binning is applied in the x-axis, then all data (x, y) where x falls inside a particular bin –i.e., a particular interval– is replaced by its bin value x_{bin} (usually the central value). Accordingly, all data falling in a given bin are coarse-grained into a single, average point. The y_{bin} is usually defined as the average of the y values for all the data falling inside that bin. Note that it is more common to find in the literature binnings in the x-axis because usually y is a dependent variable, however when both x and y are independent measurements, then one can equally bin the x-axis or the y-axis, the rule of thumb being to bin the variable which is in principle more affected by noise.

We expect high variability in the duration of words, especially when comparing words with lower frequencies together as we know that their duration comes from heavy-tailed Lognormal distributions. When binning a scatter plot of two variables where one of them has much more noise than the other, it may not be indifferent how to choose on what variable perform the binning. We present an example in Figure S8 where two variables x and y are originally related by an exponential function. It is plausible that one the variables has much uncertainty and in this case we apply a noise to variable x as it is shown in the upper right panel of Figure S8. Then we show that if binning in X-axis we do not recover the original function, while the opposite is true when binning in Y-axis.
In our work we have performed several binnings, and some of them are performed in the y-axis (frequencies). In the next section we given an intuitive explanation as of why this is the adequate choice.

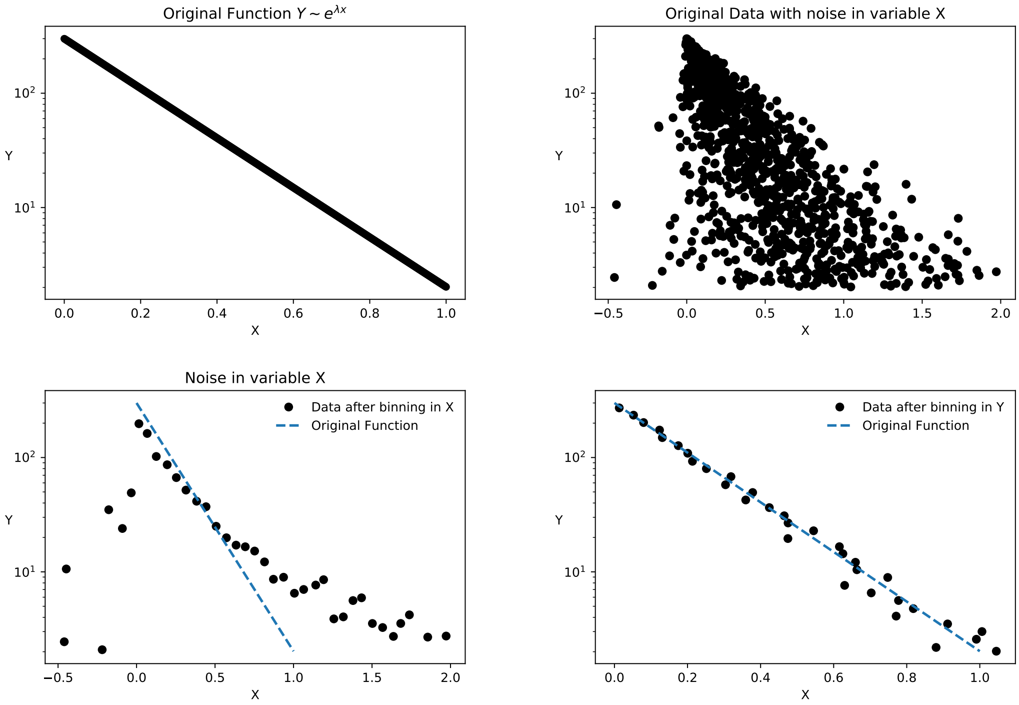

Figure S8: **Binning the y-axis.** Linear-Log plot of: (a) Upper left: Scatter plot of data generated from an exponential function $y \propto e^{\lambda x}$. (b) Upper right: Scatter plot of the original data after adding some noise to variable x. (c) Bottom left: data after binning in X-axis (black circles) and original function (blue dot line). In this way it is not possible to recover the original exponential dependence. (d) Bottom right: we recover the original function if we apply the binning into the Y-axis.

B. Binning in frequencies

Consider the main panel of figure 6 in the main manuscript, and consider all words which are only found once ($f = 1$). For each of these words, their median time duration is just equal to the time duration of the observed sample. Note however that, since words duration is lognormally distributed, individual samples will likely deviate from the average behavior. Now, let us assume now that we consider all words with frequency $f = 1$ and as the representative of the time duration of words of frequency $f = 1$, we take the average of all time durations observed. In that case, invoking the law of large numbers (the lognormal distribution has finite mean), this average will now be representative. This procedure is indeed what we achieve by making a binning by frequencies (blue dots in figure 6). Now, since according to our theory we expect an exponential relation between frequency and time duration, then such binning needs to be performed in logarithmic scales (logarithmic binning).
In other words, why is the underlying data so noisy for low frequencies in Figs 6 and 7? (something similar happens in Fig.8 for the high rank end). This is due to the fact that we are using median values for time duration, and for small frequency words (i.e. low sampling) we expect severe fluctuations due to the fact that the underlying time duration distribution is indeed Lognormal. This low sampling effect is counterbalanced, according to the law of large numbers, by making a logarithmic binning in the frequency axis for Figure 6 or in the rank axis for Figure 8.

VI. MENZERATH-ALTMANN LAW: ADDITIONAL RESULTS

A. Additional fits of MAL

In the main manuscript we provide fits of MAL to data after linear binning, here we complement those with similar fits without a linear binning, as shown in Fig.S9. All fitted parameters are reporter in table I. We confirm that MAL is only fully certified when constituent size is measured in physical units.

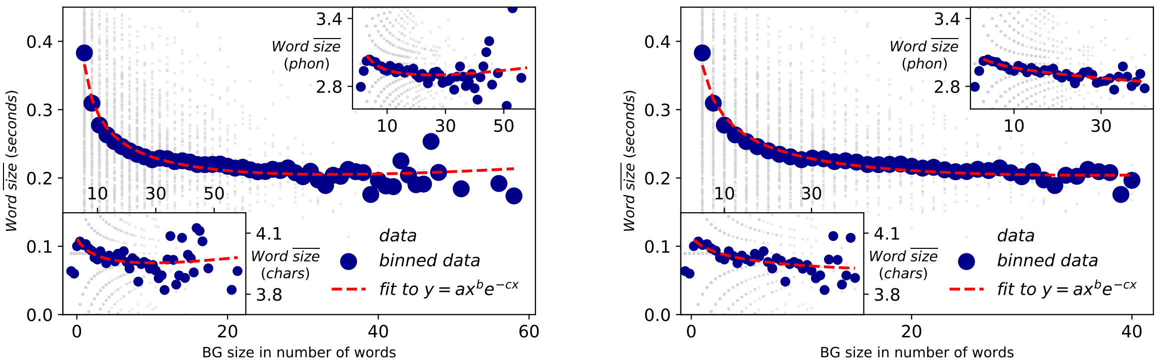

Figure S9: **Additional MAL fits.** (Left) We show the relation between BG size (measured in number of words) vs the word size, measured in time units (main panel), number of phonemes and number of characters (inset panels). Blue circles are averages over all BGs of the same size (i.e. a binning of size 1). Red dotted lines are a fit of blue circles to Menzerath-Altmann law $y(n) = an^b \exp(-cn)$ (see table I for fitting parameters). (Right panel) Similar to left panel, but where all BGs larger than 40 words have been discarded from the analysis. In both cases we confirm that MAL is only fully justified when the constituent size is measured in time units.

	a	b	c	R^2
BG vs words size (in time units), all the data	0.364	-0.227	$-6.7 \cdot 10^{-3}$	0.7
BG vs words size (in time units), only BGs<40 words	0.36	-0.22	$-6 \cdot 10^{-3}$	0.94
BG vs words (in number of phonemes > 2), all the data	3.22	$-4.7 \cdot 10^{-2}$	$-1.85 \cdot 10^{-3}$	0.05
BG vs words (in number of phonemes), all the data	2.97	$-6.8 \cdot 10^{-3}$	$-2 \cdot 10^{-4}$	0.0
BG vs words (in number of phonemes > 2), only BGs<40 words	3.12	$-2.2 \cdot 10^{-2}$	$-2.6 \cdot 10^{-4}$	0.66
BG vs words (in number of phonemes), only BGs<40 words	2.91	$-1.8 \cdot 10^{-2}$	$-2.5 \cdot 10^{-3}$	0.46
BG vs words size (in number of chars > 2), all the data	4.16	$-2.14 \cdot 10^{-2}$	$-7.4 \cdot 10^{-4}$	0.05
BG vs words size (in number of chars), all the data	3.99	$-9 \cdot 10^{-4}$	10^{-4}	0.01
BG vs words size (in number of chars > 2), only BGs<40 words	4.15	$-1.8 \cdot 10^{-2}$	$-3.7 \cdot 10^{-4}$	0.32
BG vs words size (in number of chars), only BGs<40 words	3.97	$-7.3 \cdot 10^{-3}$	$- \cdot 10^{-3}$	0.17
Words vs phoneme size (in time units), all the data	0.18	-0.23	$-7 \cdot 10^{-3}$	0.9

Table I: Parameter fits of Menzerath-Altmann's law $y(n) = an^b \exp(-cn)$ to the Buckeye corpus data for different linguistic levels (BG, words and phonemes), using all the data and only BGs up to 40 words (the law is fitted to the mean values –the blue circles in Fig.S9–). Fitting has been performed using Levenberg-Marquardt algorithm, and R^2 is used to determine the goodness of the fitting (values close to 1 indicate better fittings). MAL is only fully certified when constituent size is measured in physical units.

B. Predicting BG time duration distribution using the MAL model

Interertingly, the mathematical model that brings about MAL can also be used to predict the time distribution of breath-groups, which we already found to be approximately lognormally distributed in results section A of the main manuscript. The method goes as follows: first, we sample BGs composed of n words directly from $W(n)$ (reported in the inset plot of the right panel of Figure 3 in the main manuscript). The duration of these BGs is then predicted by the theoretical model (Eq.9 from the main text). The only pending point is how to sample $t(1)$, i.e. the time duration of the first word, which is undefined in the theoretical model. Results are shown in Figure S10. In the left panel of this figure, we randomly sample $t(1)$ directly from the empirical time distribution of words $P(t_w)$. In such a case, as we did in results section A of the main text, a small noise term with positive mean is also added systematically to mimic the VOT effect. The agreement with the empirical time distribution $P(t_{BG})$ is quite good. In the right panel of the same figure, we sample $t(1)$ directly from the actual first word time distribution (i.e., this distribution is likely to have empirically absorbed the VOT and other segmentation error effects). The resulting distribution is then in very good agreement with $P(t_{BG})$, concluding that MAL can indeed quantitatively predict BG time distribution.

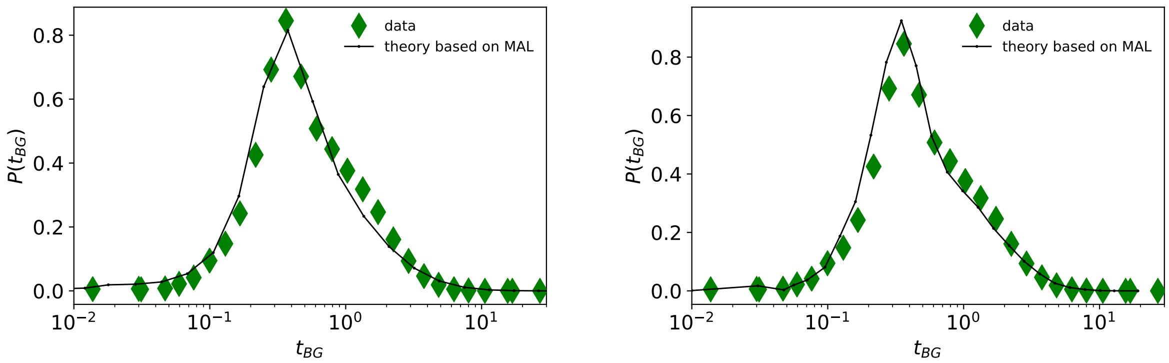

Figure S10: **BG length distribution using MAL model.** We sample BGs of n words using the real $W(n)$, and using the theoretical model Eq.9 (main text) we compute the time elapsed in each BG, thereby estimating a time distribution which can then be compared with the empirical $P(t_{BG})$. The only undefined element is how $t(1)$ (the time duration of the first word) is chosen. (Left) $t(1)$ is sampled randomly from $P(t_w)$, and a Gaussian error term with positive mean is added to each BG as in Figure 3 of main text, to model segmentation and VOT-like error effects. (Right panel) Similar to the left panel, but when $t(1)$ is sampled from real data (hence already absorbing segmentation errors). The agreement with $P(t_{BG})$ is noticeable.

---

[1] D. Zielińska, "Linguistic research in the empirical paradigm as outlined by mario bunge," *SpringerPlus*, vol. 5, p. 1183, Jul 2016.

[2] P. Grzybek, *Introductory Remarks: On the Science of Language in Light of the Language of Science*, pp. 1–13. Springer, 2006.

[3] T. Grabinska and D. Zielinska, "Linguistics from the perspective of the theory of models in empirical sciences: From formal to corpus linguistics," *Journal of Technical Writing and Communication*, vol. 40, no. 4, pp. 379–402, 2010.

[4] T. F. T. F. Quatieri, *Discrete-time speech signal processing : principles and practice*. Upper Saddle River, N.J. ; London : Prentice Hall PTR, 2002.

[5] M. A. Pitt, K. Johnson, E. Hume, S. Kiesling, and W. Raymond, "The buckeye corpus of conversational speech: labeling conventions and a test of transcriber reliability," *Speech Communication*, vol. 45, no. 1, pp. 89–95, 2005.

[6] M. A. Pitt, L. Dilley, K. Johnson, S. Kiesling, W. Raymond, E. Hume, and E. Fosler-Lussier, "Buckeye corpus of conversational speech (2nd release)[www. buckeyecorpus. osu. edu] columbus, oh: Department of psychology," *Ohio State University (Distributor)*, 2007.

[7] S. Greenberg, H. Carvey, L. Hitchcock, and S. Chang, "Temporal properties of spontaneous speech-a syllable-centric perspective," *Journal of Phonetics*, vol. 31, no. 3-4, pp. 465–485, 2003.

[8] C. E. Shannon, "Prediction and entropy of printed english," *Bell System Technical Journal*, vol. 30, pp. 50–64, Jan. 1951.

[9] O. Crouzet, "On segments and syllables in the sound structure of language: curve-based approaches to phonology and the auditory representation of speech," *Mathématiques et sciences humaines*, vol. 180, no. 4, pp. 57–71, 2007.

[10] S. Greenberg, H. Carvey, L. Hitchcock, and S. Chang, "Temporal properties of spontaneous speech—a syllable-centric perspective," *Journal of Phonetics*, vol. 31, no. 3-4, pp. 465–485, 2003.

[11] Y.-T. Wang, J. R. Green, I. S. Nip, R. D. Kent, and J. F. Kent, "Breath Group Analysis for Reading and Spontaneous Speech in Healthy Adults," *Folia Phoniatrica et Logopaedica*, vol. 62, pp. 297–302, 06 2010.

[12] R. E. Hayden, "The relative frequency of phonemes in general-american english," *WORD*, vol. 6, no. 3, pp. 217–223, 1950.

[13] N. Umeda, "Consonant duration in american english," *The Journal of the Acoustical Society of America*, vol. 61, no. 3, pp. 846–858, 1977.

[14] N. Umeda, "Vowel duration in american english," *The Journal of the Acoustical Society of America*, vol. 58, no. 2, pp. 434–445, 1975.

[15] W. Hardcastle and N. Hewlett, *Coarticulation: Theory, Data and Techniques*. Cambridge Studies in Speech Science and Communication, Cambridge University Press, 2006.

[16] B. Mandelbrot, "On the theory of word frequencies and on related markovian models of discourse," in *Structure of Language and its Mathematical Aspects* (R. Jakobson, ed.), vol. XII, pp. 190–210, American Mathematical Society, 1961.

[17] G. A. Miller, "Some effects of intermittent silence," *The American Journal of Psychology*, vol. 70, no. 2, pp. 311–314, 1957.

[18] R. Ferrer-i Cancho and B. Elvevåg, "Random texts do not exhibit the real zipf's law-like rank distribution," *PLOS ONE*, vol. 5, pp. 1–10, 03 2010.

[19] R. Baayen, *Word Frequency Distributions*. Kluwer Academic Publishers, Springer, 2001.

[20] S. T. Piantadosi, "Zipf's word frequency law in natural language: A critical review and future directions," *Psychonomic Bulletin & Review*, vol. 21, pp. 1112–1130, Oct 2014.

[21] J. R. Williams, J. P. Bagrow, C. M. Danforth, and P. S. Dodds, “Text mixing shapes the anatomy of rank-frequency distributions,” *Physical Review E*, vol. 91, no. 5, p. 052811, 2015.
